# Household economic impact of HIV‐associated cryptococcal meningitis in five countries in Southern and Eastern Africa

**DOI:** 10.1002/jia2.26441

**Published:** 2025-06-05

**Authors:** David S. Lawrence, Charles Muthoga, Jack Adams, Antoinette Buhle Ndweni, David R. Boulware, Chimwemwe Chawinga, Kyla Comins, Eltas N. Dziwani, Admire Hlupeni, Mina C. Hosseinipour, Samuel Jjunju, Cecilia Kanyama, Tshepo B. Leeme, Graeme Meintjes, David B. Meya, Mosepele Mosepele, Melanie Moyo, Henry C. Mwandumba, Conrad Muzoora, Chiratidzo E. Ndhlovu, Edwin Nuwagira, Charlotte Schutz, Lillian Tugume, Darlisha Williams, Síle F. Molloy, Timothée Boyer‐Chammard, Nabila Youssouf, Shabbar Jaffar, Louis W. Niessen, Thomas S. Harrison, Lucy Cunnama, Joseph N. Jarvis, the AMBITION Study Group

**Affiliations:** ^1^ Clinical Research Department Faculty of Infectious and Tropical Diseases London School of Hygiene and Tropical Medicine London UK; ^2^ Botswana Harvard Health Partnership Gaborone Botswana; ^3^ Institute for Infection and Immunity St George's University London London UK; ^4^ Health Economics Unit and Division School of Public Health Health Sciences Faculty University of Cape Town Cape Town South Africa; ^5^ Infectious Diseases Institute College of Health Sciences Makerere University Kampala Uganda; ^6^ University of Minnesota Minneapolis Minnesota USA; ^7^ Lilongwe Medical Relief Trust (UNC Project) Lilongwe Malawi; ^8^ Wellcome Centre for Infectious Diseases Research in Africa (CIDRI‐Africa) Institute of Infectious Disease and Molecular Medicine University of Cape Town Cape Town South Africa; ^9^ Malawi‐Liverpool‐Wellcome Clinical Research Programme Blantyre Malawi; ^10^ Internal Medicine Department Faculty of Medicine and Health Sciences University of Zimbabwe Harare Zimbabwe; ^11^ Department of Medicine University of North Carolina Chapel Hill North Carolina USA; ^12^ Department of Medicine University of Cape Town Cape Town South Africa; ^13^ Department of Medicine School of Medicine Makerere University Kampala Uganda; ^14^ Department of Internal Medicine University of Botswana Gaborone Botswana; ^15^ Department of Medicine Kamuzu University of Health Sciences Blantyre Malawi; ^16^ Mbarara University of Science and Technology Mbarara Uganda; ^17^ Institut Pasteur CNRS Molecular Mycology Unit and National Reference Center for Invasive Mycoses and Antifungals, UMR 2000 Paris France; ^18^ Institute for Global Health University College London London UK; ^19^ Department of Public Health and Clinical Sciences, Liverpool School of Tropical Medicine Liverpool UK; ^20^ Department of International Health Johns Hopkins School of Public Health Baltimore Maryland USA; ^21^ Clinical Academic Group in Infection and Immunity St George's University Hospitals NHS Foundation Trust London UK; ^22^ MRC Centre for Medical Mycology University of Exeter Exeter UK

**Keywords:** HIV, cryptococcal meningitis, cost analysis, out‐of‐pocket expenditure, catastrophic healthcare expenditure, clinical trial

## Abstract

**Introduction:**

HIV‐associated cryptococcal meningitis is the second leading cause of AIDS‐related mortality. Cryptococcal meningitis is a poverty‐related disease and the majority of cases occur in settings where resources are limited and access to quality care is often linked to an individual's ability to pay for services. We have previously demonstrated the efficacy, safety and cost‐effectiveness of a single, high‐dose liposomal amphotericin‐based treatment regimen within the AMBITION‐cm trial. Here, we present a five‐country, within‐trial analysis exploring the household economic impact of cryptococcal meningitis.

**Methods:**

Eight hundred and ten participants were recruited into this sub‐study in Botswana, Malawi, South Africa, Uganda and Zimbabwe between January 2018 and February 2021. We collected data on annual household expenditure, direct costs and indirect costs incurred prior to enrolment and during the 10‐week trial period. Costs were inflated and converted to 2022 USD. We calculated out‐of‐pocket expenditure, lost income and catastrophic healthcare expenditure, defined as costs exceeding 20% of annual household expenditure.

**Results:**

The average total out‐of‐pocket expenditure plus lost income prior to enrolment was $132 and 17.9% (145/810, 95% CI 15.3–20.5) of participant households had already experienced catastrophic healthcare expenditure. Among the 592 surviving participants, when combining out‐of‐pocket expenditure and lost income, the average cost was $516 and 29.1% of annual household expenditure across all countries, ranging from $230 (7.6%) in South Africa to $592 (64.2%) in Zimbabwe. More than half (296/581, 51.0%, 95% CI 46.9–55.0) of households experienced catastrophic healthcare expenditure by the end of the trial, ranging from 16.0% (13/81, 95% CI 7.9–24.2) in South Africa to 68.1% (156/229, 95% CI 62.0–74.2) in Uganda.

**Conclusions:**

This is the first study exploring the household economic impact experienced by those diagnosed with cryptococcal meningitis. The household economic impact of cryptococcal meningitis is high and more than half of households of individuals who survive experience catastrophic healthcare expenditure. It is likely these figures are higher outside of the research setting. This highlights the profound financial impact of this devastating infection and provides a rationale to offer financial and social protection to those affected.

**Trial Registration Number:**

ISRCTN72509687

## 1 INTRODUCTION

HIV‐associated cryptococcal meningitis is the second‐leading cause of AIDS‐related mortality and is responsible for approximately 19% of AIDS‐related deaths worldwide [1]. Cryptococcal meningitis is a poverty‐related disease and most cases occur in sub‐Saharan Africa where resources are limited and access to quality care is often linked to an ability to pay [2]. Governments may partially or fully fund direct costs related to hospital admissions and outpatient management but the individual and their households, family and friends also incur out‐of‐pocket expenses. The World Health Organization (WHO) acknowledges that progress towards Universal Health Coverage as a core Sustainable Development Goal (SDG) can only be achieved if all can obtain the health services they need without suffering financial hardship and with financial risk protection (SDG Target 3.8) [3, 4]. However, in Africa, at least 37% of healthcare spending is out‐of‐pocket expenditure [5] resulting in a high financial burden on those with lower incomes [6].

Catastrophic healthcare expenditure (CHE) has been defined as out‐of‐pocket expenditure above a proportion of total household expenditure which may be associated with households sacrificing other essentials such as food, incurring debt and can lead to impoverishment [7]. There are multiple proportions, or thresholds, used in the definition of CHE ranging from 10% to 25% of annual household expenditure [7, 8]. A systematic review and meta‐analysis in sub‐Saharan Africa using a 10% threshold found a pooled annual incidence of 16.5% for all illnesses (95% confidence interval [CI] 12.9–20.4; 50 datapoints; *I* ^2^ = 99.9%) and an incidence for HIV‐related illness of 27.1% (95% CI 15.6–40.5; 3 datapoints; *I* ^2^ = 98.7%) [9]. CHE at a threshold of 10% has been reported to be as high as 100% for HIV‐related hospital admissions in some settings [10].

The high levels of CHE for HIV‐related illness have been attributed to higher costs when seeking care, which can often involve numerous healthcare interactions, prolonged hospital admissions, and extensive non‐medical expenses such as travel and food [9]. Cryptococcal meningitis typically presents with a headache that becomes more debilitating over days and weeks. During this time, individuals typically visit numerous different healthcare facilities as their symptoms worsen, many of which are private providers, and often transition back to the public sector as they deteriorate and require hospitalization [11]. This contributes to being admitted and diagnosed with more severe cryptococcal meningitis. Those diagnosed at the point where they have developed confusion due to severe meningitis have more than twice the mortality as those without confusion, so these delays contribute to worse outcomes [12, 13].

Cryptococcal meningitis is diagnosed by lumbar puncture and treatment is with a combination of antifungals administered in hospital. Additional lumbar punctures are often required to manage increased pressure around the brain, a common complication. Antifungal treatment has previously been based on 7‐ to 14‐day courses of intravenous amphotericin B deoxycholate which is associated with drug‐related toxicities and prolonged hospital admissions, leading to higher costs [14]. The AMBIsome Therapy Induction Optimisation (AMBITION‐cm) trial was a phase‐III non‐inferiority trial comparing a single, high‐dose, intravenous liposomal amphotericin (L‐AmB)‐based regimen with the previous WHO recommended regimen based on seven daily doses of amphotericin B deoxycholate [12]. Based on the trial results, the WHO updated their guidelines in 2022 to recommend the single high‐dose L‐AmB regimen as first‐line therapy [15]. A within‐trial cost‐effectiveness analysis across the five trial country settings found the regimen to be highly cost‐effective with incremental cost‐effectiveness ratios ranging from USD (United States Dollars) $71 in Botswana to $121 in Uganda per life‐year saved [16].

To date, there have been no studies exploring out‐of‐pocket expenditure and CHE experienced by the households of individuals with cryptococcal meningitis. We embedded a patient cost study within AMBITION‐cm with the aim of describing the household economic impact of cryptococcal meningitis.

## 2 METHODS

### 2.1 The AMBITION‐cm trial

The AMBITION‐cm trial has been described above and in detail elsewhere [12, 17] and a dedicated protocol for this economic analysis is available [18]. A total of 844 participants with HIV‐associated cryptococcal meningitis were enrolled from eight hospitals in five countries (Botswana, Malawi, South Africa, Uganda and Zimbabwe) between January 2018 and February 2021. The proportion who died at 10 weeks was 24.8% in the L‐AmB arm compared to 28.7% in the control arm. The regimen was non‐inferior in the unadjusted analysis, superior in the adjusted analysis, associated with fewer grade 3 or 4 adverse events (50.0% vs. 62.3%) and highly acceptable to both participants and healthcare workers [19].

### 2.2 Baseline data

At baseline, each participant completed an interviewer‐administered questionnaire with study staff (Table S1) [18]. The questionnaire solicited demographic information and asked participants how much their household typically spent on food per week, rent and utilities per month, and large purchases (e.g. furniture, electrical items, cars) in the last year. We did not ask about absolute household income, an active decision to avoid potentially infringing on the participant's privacy. We asked how much money they and/or someone else had spent on activities related to their health in the 4 weeks prior to being recruited into the trial to capture most costs while limiting recall bias. We asked about the cost and time spent on travel to the hospital for their admission and previous interactions with healthcare facilities prior to admission. We asked for up to three of the most recent healthcare encounters to balance the need for in‐depth information with recall bias and responder fatigue, particularly given the severity of their infection. We asked about access and use of private insurance and financial coping mechanisms such as taking out loans or selling possessions to pay for healthcare. In participants with confusion, we waited several days to collect the data should their recall improve. If this was not possible, we obtained data from their next‐of‐kin.

### 2.3 End of study data

At the end of the 10‐week trial, survivors contributed to a shorter interviewer‐delivered questionnaire to understand how long they had been unwell for, how much work they had missed and any lost income, as well as other out‐of‐pocket expenditure. The trial provided travel reimbursements and medical care throughout the 10‐week follow‐up period so additional expenses were expected to be low. Loss of income for caregivers was not captured. For those who died, we did not collect data on costs related to funerals and persistent loss of income to avoid distressing the bereaved.

### 2.4 Analysis

Data are presented overall and by country. Demographic data were described. Occupations were classified in line with the International Standard Classification of Occupations (ISCO‐08) [20]. We summarized previous healthcare interactions. Costing data are presented in 2022 USD. Costs were adjusted for consumer price index inflation (sourced from the World Bank) based on the year of recruitment and converted to USD.

The economic analysis is presented across two cohorts. The first is all participants and details expenditure and lost income in the 4 weeks prior to enrolment. The second is only those who survived to 10 weeks and could provide end‐of‐study data. We multiplied weekly food expenses by 52 and monthly rent and utilities by 12 and added these to annual larger expenses to generate an estimated annual household expenditure. In line with WHO tuberculosis (TB) patient cost surveys, we defined CHE as out‐of‐pocket expenditure and lost income of at least 20% of annual household expenditure and calculated for both cohorts [21]. All analyses were conducted using STATA SE v15·1.

### 2.5 Sensitivity analysis

As the definition of CHE varies in the literature, we also calculated this using the threshold of 10% of annual household expenditure. We also performed analyses by gender and treatment arm, comparing means using a *t*‐test and defining statistical significance as *p* <0.05. The final sensitivity analysis relates to the currencies used in Zimbabwe. During the trial, both USD and Zimbabwean dollars (ZWD) were used interchangeably, the latter of which was subject to intense exchange rate volatility and inflation. We conducted an exploratory analysis of the cost to households based on which currency they used. Costs incurred by households who paid with ZWD were adjusted and converted to USD and compared directly with those incurred by households who paid with USD, which were adjusted as necessary.

### 2.6 Ethical considerations

The protocol was approved by research ethics committees at the London School of Hygiene and Tropical Medicine, Botswana Ministry of Health and Wellness, Malawi National Health Sciences, University of Cape Town, Uganda National Council for Science and Technology, and Zimbabwe Medical Research Council. Written informed consent was obtained from participants or from the next‐of‐kin if participants were incapable of consenting. If a participant recovered capacity, written informed consent was obtained from them and they were free to leave the study if they wished.

## 3 RESULTS

### 3.1 Study population

A total of 844 participants were recruited into AMBITION‐cm with 814 included in the trial intention‐to‐treat analysis. Four withdrew consent for further studies and did not provide economic data, leaving 810 participants included in this analysis (73 in Botswana; 230 in Malawi; 106 in South Africa; 330 in Uganda; and 71 in Zimbabwe) (Table 1); 39% (319/810) were female and the median age was 37 years (IQR 32–43 years). The level of education was similar across countries except Uganda where most participants had not attended secondary school. The trial participant was the main earner in 70% (562/808) of households and the average annual expenditure was $1717 (SD $1939) per household. This varied across countries with household expenditure higher in Botswana and South Africa and lower in Zimbabwe.

**Table 1:** Baseline demographics and household expenditure

Participants	Overall	Botswana	Malawi	South Africa	Uganda	Zimbabwe
	*n* = 810	*n* = 73	*n* = 230	*n* = 106	*n* = 330	*n* = 71
**Gender (*n* [%])**						
Female	319 (39.4)	19 (26.0)	86 (37.4)	59 (55.7)	130 (39.4)	25 (35.2)
Male	491 (60.6)	54 (74.0)	144 (62.6)	47 (44.3)	200 (60.6)	46 (64.8)
**Age, years**						
Median (IQR)	37 (32–43)	39 (34–44)	38 (32–44)	37 (32–44)	35 (30–40)	39 (33–45)

	***n* = 808**	***n* = 72**		***n* = 105**		
**Years in education (*n* [%])**						
Median (IQR)	10 (7–12)	11 (9–12)	10 (7–12)	11 (9–12)	7 (6–11)	11 (9–13)
0	28 (3.5)	1 (1.4)	3 (1.3)	3 (2.9)	20 (6.1)	1 (1.4)
1–7	263 (32.5)	9 (12.5)	66 (28.7)	15 (14.3)	163 (49.4)	10 (14.1)
8–12	382 (47.3)	49 (68.1)	115 (50.0)	79 (75.2)	100 (30.3)	39 (54.9)
>12	135 (16.7)	13 (18.1)	46 (20.0)	8 (7.6)	47 (14.2)	21 (29.6)

	***n* = 808**	***n* = 72**		***n* = 105**		
**Highest qualification (*n* [%])**						
None	126 (15.6)	1 (1.4)	64 (27.8)	10 (9.5)	50 (15.2)	1 (1.4)
Primary school certificate	227 (28.1)	8 (11.1)	46 (20.0)	12 (11.4)	151 (45.8)	10 (14.1)
Secondary school certificate	326 (40.3)	51 (70.8)	74 (32.2)	77 (73.3)	82 (24.9)	42 (59.2)
Diploma	109 (13.5)	10 (13.9)	37 (16.1)	6 (5.7)	39 (11.8)	17 (23.9)
Undergraduate degree	14 (1.7)	1 (1.4)	5 (2.2)	0 (0)	7 (2.1)	1 (1.4)
Postgraduate degree	6 (0.7)	1 (1.4)	4 (1.7)	0 (0)	1 (0.3)	0 (0)
**Occupation (*n* [%])**						
Legislators, senior officials and managers	6 (0.74)	0 (0)	2 (0.9)	1 (0.9)	2 (0.6)	1 (1.4)
Professionals	57 (7.04)	5 (6.8)	22 (9.6)	5 (4.7)	16 (4.8)	9 (12.7)
Technicians and associate professionals	37 (4.57)	11 (15.1)	3 (1.3)	1 (0.9)	17 (5.2)	5 (7.0)
Clerks	6 (0.74)	0 (0)	3 (1.3)	1 (0.9)	1 (0.3)	1 (1.4)
Service, shop and market sales workers	266 (32.84)	16 (21.9)	85 (37.0)	22 (20.8)	130 (39.4)	13 (18.3)
Skilled agricultural and fishery workers	31 (3.83)	0 (0)	8 (3.5)	1 (0.9)	20 (6.1)	2 (2.8)
Craft and related trades workers	100 (12.35)	12 (16.4)	23 (1.0)	10 (9.4)	47 (14.2)	8 (11.3)
Plant and machinery operators	3 (0.37)	3 (4.1)	0 (0)	0 (0)	0 (0)	0 (0)
Elementary occupations	141 (17.41)	9 (12.3)	27 (11.7)	29 (27.4)	63 (19.1)	13 (18.3)
Armed forces	57 (7.04)	6 (8.2)	34 (14.8)	13 (12.3)	2 (0.6)	2 (2.8)
Occupations unspecified and not economically active persons	106 (13.09)	11 (15.1)	23 (10.0)	23 (21.7)	32 (9.7)	17 (23.9)

	***n* = 808**	***n* = 72**		***n* = 105**		
**Participant main earner in household (*n* [%])**						
Yes	562 (69.6)	57 (79.2)	147 (63.9)	60 (57.1)	248 (75.2)	50 (70.4)
No	246 (30.4)	15 (20.8)	83 (36.1)	45 (42.9)	82 (24.8)	21 (29.6)
**Weekly food expenditure (Mean [SD]) USD**	23.44 (27.09)	29.03 (27.23)	21.82 (27.11)	44.82 (36.38)	18.95 (22.04)	11.91 (9.28)
**Monthly rent and utilities (Mean [SD]) USD**	30.38 (53.83)	71.91 (58.46)	31.13 (58.84)	35.72 (90.06)	23.21 (28.33)	10.54 (15.09)
**Annual large purchases (Mean [SD]) USD**	133.39 (644.70)	340.47 (1025.85)	52.70 (400.51)	103.98 (323.39)	150.07 (771.18)	148.18 (418.88)
**Estimated annual expenditure (Mean [SD]) USD**	1716.93 (1939.14)	2713.10 (2072.41)	1561.05 (1918.62)	2863.13 (2744.96)	1414.08 (1516.95)	894.04 (810.42)

*Note*: Data were missing for two participants, one in Botswana and one in South Africa.

Abbreviations: IQR, interquartile range; SD, standard deviation; USD, United States Dollar.

### 3.2 Costs incurred prior to hospitalization

In the 4 weeks prior to enrolment, participants reported headache symptoms for a median of 14 days (IQR 7–24) (Table 2). Seventy‐eight percent of participants (634/810) had missed work, with 53% (338/634) of those losing an average of $162 (SD $300) in income. A higher proportion of individuals were economically inactive in South Africa (35%, 37/106) than in the other country settings. Other caregivers had provided support for a median of 2 days (IQR 0–7 days) with this highest in Uganda (median 5 days [IQR 2–14 days]).

**Table 2:** Direct and indirect costs incurred due to cryptococcal meningitis

In the 4 weeks prior to enrolment:	Overall	Botswana	Malawi	South Africa	Uganda	Zimbabwe
	*n* = 810	*n* = 73	*n* = 230	*n* = 106	*n* = 330	*n* = 71
Duration of illness in days						
Median (IQR)	14 (7–24)	7 (4–14)	14 (7–21)	14 (7–21)	14 (10–30)	14 (9–21)
Mean (SD)	19.5 (19.5)	10.9 (10.5)	19.0 (17.3)	17.4 (16.1)	23.1 (23.7)	16.8 (10.9)
Primary activity missed due to illness (*n* [%])						
Working	634 (78.3%)	59 (80.8%)	177 (77.0%)	54 (50.9%)	288 (87.3%)	56 (78.9%)
Studying	17 (2.1%)	0	11 (4.8%)	1 (0.9%)	4 (1.2%)	1 (1.4%)
Maintaining the house	28 (3.5%)	2 (2.7%)	4 (1.7%)	9 (8.5%)	9 (2.7%)	4 (5.6%)
Caring for children	35 (4.3%)	1 (1.4%)	20 (8.7%)	5 (4.7%)	2 (0.6%)	7 (9.9%)
Nothing	96 (11.9%)	11 (15.1%)	18 (7.8%)	37 (34.9%)	27 (8.2%)	3 (4.2%)

	***n* = 634**	***n* = 59**	***n* = 117**	***n* = 54**	***n* = 288**	***n* = 56**
If working, days spent off work						
Median (IQR)	14 (7–21)	6 (3–11)	14 (7–21)	11 (6–20)	14 (7–30)	9 (7–20)
Mean (SD)	17.4 (19.3)	8.0 (8.9)	17.4 (15.8)	15.5 (16.0)	20.4 (23.4)	13 (10.6)

	***n* = 634**	***n* = 59**	***n* = 177**	***n* = 54**	***n* = 288**	***n* = 56**
If working, lost income before trial (*n* [%])						
Yes	338 (53.3%)	20 (33.9%)	74 (41.8%)	19 (35.2%)	191 (66.3%)	34 (60.7%)
No	296 (46.7%)	39 (66.1%)	103 (58.2%)	35 (64.8%)	97 (33.7%)	22 (39.3%)

	***n* = 338**	***n* = 20**	***n* = 74**	***n* = 19**	***n* = 191**	***n* = 34**
If working, income lost before trial (Mean [SD]) USD	162.31 (299.83)	306.77 (351.71)	166.43 (291.57)	211.71 (244.75)	158.82 (321.63)	**60.35** (75.34)
Days others spent providing care before trial						
Median (IQR)	2 (0–7)	0 (0–0)	2 (1–8)	0 (0–0)	5 (2–14)	2 (2–5)
Mean (SD)	6.2 (11.2)	0.3 (0.9)	6.8 (10.4)	0.2 (0.9)	9.5 (13.7)	4.4 (7.2)
Number of previous healthcare interactions (Median [IQR])	1 (1–2)	1 (0–1)	1 (1–2)	1(1–2)	1(1–2)	1 (1–1)
Overall cost of previous healthcare interactions (Mean [SD])	27.13 (56.83)	19.88 (99.97)	24.22 (52.90)	18.50 (33.45)	35.09 (55.84)	19.88 (31.77)
Personal expenditure (Mean [SD]) USD	36.64 (73.28)	25.58 (100.78)	38.69 (74.64)	19.64 (31.87)	46.21 (78.55)	22.29 (40.54)
Expenditure of others (Mean [SD]) USD	28.06 (65.26)	5.24 (11.85)	31.28 (64.93)	2.22 (7.35)	36.70 (59.94)	39.55 (127.48)
**Overall out of pocket expenditure (Mean [SD]) USD**	64.71 (103.78)	30.83 (100.92)	69.97 (109.43)	21.86 (32.93)	82.92 (103.90)	61.84 (129.67)
**Overall out of pocket expenditure plus lost income (Mean [SD]) USD**	132.44 (250.02)	114.87 (280.04)	123.53 (231.25)	59.81 (134.97)	174.84 (291.38)	90.74 (147.07)
Access to private healthcare insurance (*n* [%])						
Yes	20 (2.5%)	1 (1.4%)	8 (3.5%)	0 (0%)	1 (0.3%)	10 (14.1%)
No	790 (97.5%)	72 (98.6%)	222 (96.5%)	106 (100%)	329 (99.7%)	61 (85.9%)
Used private healthcare insurance (*n* [%])						
Yes	15 (75%)	1 (100%)	5 (62.5%)	N/A	0 (0%)	9 (90.0%)
No	5 (25%)	0 (0%)	3 (37.5%)	N/A	1 (100%)	1 (10.0%)
Borrowed money (*n* [%])						
Yes	84 (10.4%)	1 (1.4%)	30 (13.0%)	3 (2.8%)	32 (9.7%)	18 (25.4%)
No	726 (89.6%)	72 (98.6%)	200 (87.0%)	103 (97.2%)	298 (90.3%)	53 (74.6%)
Sold possessions (*n* [%])						
Yes	49 (6.0%)	1 (1.4%)	22 (9.6%)	2 (1.9%)	20 (6.1%)	4 (5.6%)
No	761 (94.0%)	72 (98.6%)	208 (90.4%)	104 (98.1%)	310 (93.9%)	67 (94.4%)

**In the 4 weeks before and 10 weeks of the trial**	***n* = 581**	***n* = 60**	***n* = 163**	***n* = 81**	***n* = 229**	***n* = 48**
Overall duration of illness						
Median (IQR)	77 (60–90)	73 (47–82)	77 (44–84)	84 (77–91)	82 (65–100)	67 (40–79)
Mean (SD)	75.2 (28.9)	65.7 (25.6)	70.7 (28.2)	82.3 (16.3)	81.5 (31.9)	60.2 (25.5)
Primary activity missed due to illness						
Working	455 (78.3%)	49 (81.7%)	125 (76.7%)	34 (42.0%)	206 (90.0%)	41 (85.4%)
Studying	16 (2.8%)	0 (0%)	9 (5.5%)	1 (1.2%)	5 (2.2%)	1 (2.1%)
Maintaining the house	15 (2.6%)	1 (1.7%)	4 (2.5%)	3 (3.7%)	5 (2.2%)	2 (4.2%)
Caring for children	23 (4.0%)	1 (1.7%)	12 (7.4%)	3 (3.7%)	6 (2.6%)	1 (2.1%)
Nothing	72 (12.4%)	9 (15.0%)	13 (8.0%)	40 (49.4%)	7 (3.1%)	3 (6.3%)

	***n* = 455**	***n* = 49**	***n* = 125**	***n* = 34**	***n* = 206**	***n* = 41**
Total days off work						
Median (IQR)	73 (50–84)	68 (45–77)	75 (42–84)	76 (58–84)	77 (59–88)	62 (31–78)
Mean (SD)	69.1 (30.3)	60.9 (23.9)	65.0 (26.5)	68.9 (26.0)	76.1 (33.1)	55.2 (27.1)

	***n* = 455**	***n* = 49**	***n* = 125**	***n* = 34**	***n* = 206**	***n* = 41**
If working, lost income during trial (*n* [%])						
Yes	326 (71.7%)	25 (51%)	58 (46.4%)	22 (64.7%)	191 (92.7%)	30 (73.2%)
No	129 (28.3%)	24 (49%)	67 (53.6%)	12 (35.3%)	15 (7.3%)	11 (26.8%)

	***n* = 455**	***n* = 49**	***n* = 125**	***n* = 34**	***n* = 206**	***n* = 41**
If working and lost income, total lost income (Mean [SD]) USD	558.97 (2064.25)	720.01 (715.72)	967.25 (4639.61)	580.65 (516.78)	383.36 (505.25)	737.56 (1600.71)
Total days others spent providing care						
Median (IQR)	17 (1–30)	0 (0–2)	24 (16–51)	0 (0–0)	23 (16–41)	13 (10–18)
Mean (SD)	24.1 (27.0)	3.3 (10.6)	33.7 (27.1)	0.5 (2.1)	32.8 (28.6)	15.7 (11.0)
Overall out of pocket expenditure before and during trial (Mean [SD]) USD	132.23 (173.34)	47.31 (97.58)	190.58 (178.49)	26.74 (38.33)	155.90 (199.15)	105.38 (102.42)
**Overall out of pocket expenditure plus lost income (Mean [SD]) USD**	516.08 (1633.16)	396.58 (646.44)	589.97 (2834.74)	230.15 (488.37)	579.99 (682.53)	592.12 (1349.98)
Access to private healthcare insurance (*n* [%])						
Yes	21 (3.6%)	3 (5.0%)	10 (6.1%)	0 (0%)	2 (0.9%)	6 (12.5%)
No	560 (96.4%)	57 (95.0%)	153 (93.9%)	81 (100%)	227 (99.1%)	42 (87.5%)
Used private healthcare insurance (*n* [%])						
Yes	9 (42.9%)	0 (0%)	5 (50.0%)	N/A	0 (0%)	4 (66.6%)
No	12 (57.1%)	3 (100%)	5 (50.0%)	N/A	2 (100%)	2 (33.3%)
Borrowed money (*n* [%])						
Yes	75 (12.9%)	1 (98.3%)	24 (14.7%)	3 (3.7%)	35 (15.3%)	12 (25.0%)
No	506 (87.1%)	59 (1.7%)	139 (85.3%)	78 (96.3%)	194 (84.7%)	36 (75.0%)
Sold possessions (*n* [%])						
Yes	65 (11.2%)	2 (3.3%)	18 (11.0%)	3 (96.3%)	35 (15.3%)	7 (14.6%)
No	516 (88.8%)	58 (96.7%)	145 (89.0%)	80 (3.7%)	194 (84.7%)	41 (85.4%)

**Figure 1:**
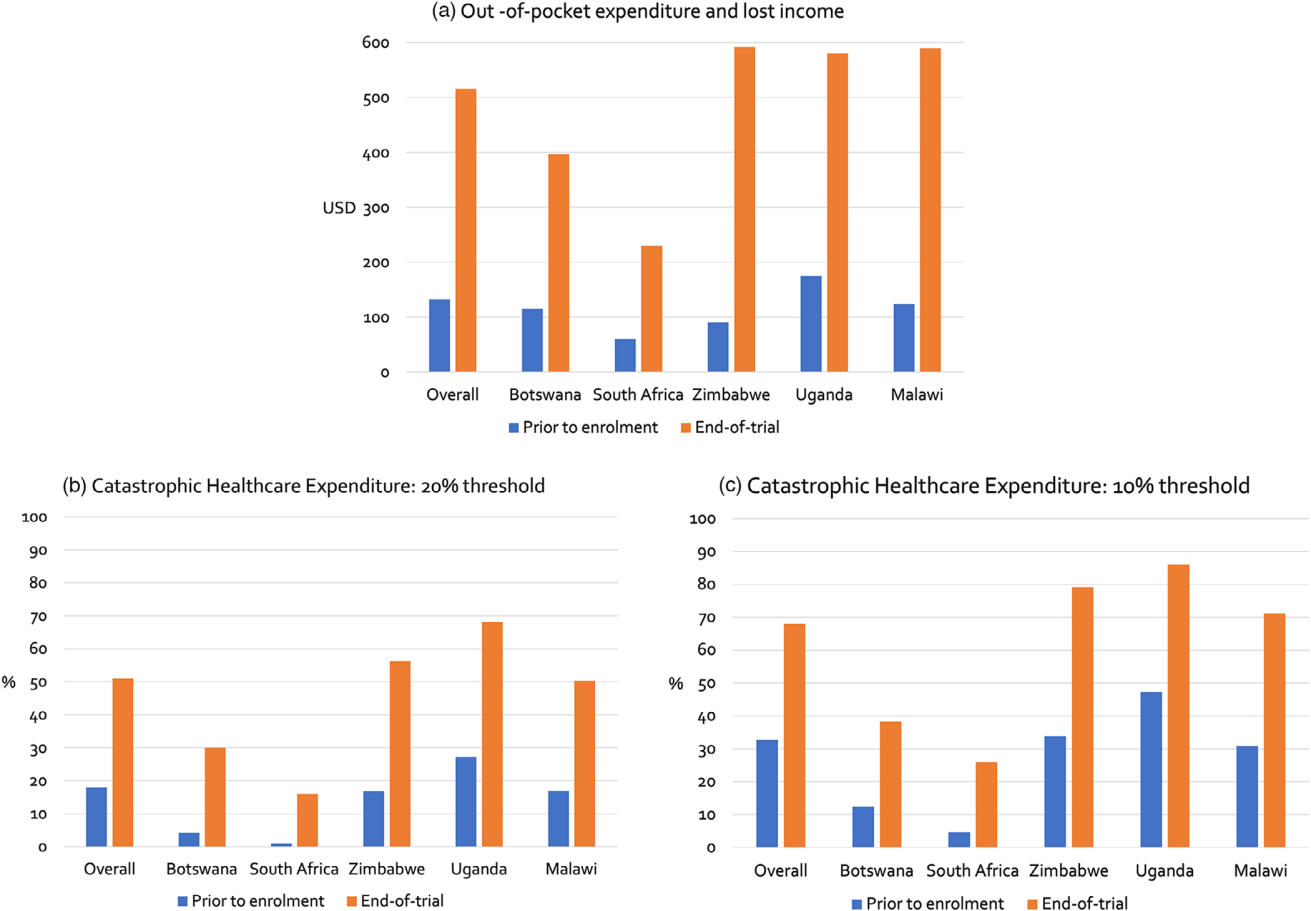
(A) Out‐of‐pocket expenditure and lost income in United States Dollars, (B) catastrophic healthcare expenditure calculated using a threshold of 20% of annual household expenditure and (C) 10% of annual household expenditure. Results are presented overall and by country, organized by decreasing gross domestic product per capita, with bars representing all participants prior to enrolment into the trial (left) and those who survived the 10‐week trial (right).

Participants had visited another healthcare facility for care on a median of one occasion (IQR 1–2) prior to hospitalization, costing an average of $27 (SD $57). When combining all costs related to their illness in the 4 weeks prior to hospitalization, participants had spent on average $37 (SD $73) of their own money and $28 (SD $65) of money from others, a total of $65 (SD $104). This varied from $22 to $83 across countries, being lowest in South Africa and highest in Uganda. The average total out‐of‐pocket expenditure plus lost income was $132 (SD $250), and this was highest in Uganda ($175) (Figure 1A). Only 2.5% (20/810) had private healthcare insurance and the majority (15/20) had accessed this. Ten percent (84/810) of all participants had borrowed money and 6% (49/810) had sold possessions to pay for care.

### 3.3 Costs incurred during the trial

A total of 592 participants survived the 10‐week trial period and economic data were available for 581. Data related to the 10‐week trial period alone are shown in Table S2. When combining the baseline and end‐of‐trial data, the surviving cohort had been unwell for a median duration of 77 days (IQR 60–90 days) (Table 2). The 78% (455/581) who were working missed a median of 73 days (IQR 50–84 days) of work. Of those who missed work, 72% (326/455) lost an average of $559 (SD $2064) in income. Participants had care provided by others for a median of 17 days (IQR 1–30 days), with this being higher in Malawi, Uganda and Zimbabwe. Among all survivors, the average out‐of‐pocket expenditure due to their illness was $132 (SD $173), and this was highest in Uganda ($156) and Malawi ($191). When combining out‐of‐pocket expenditure and lost income, the average cost was $516 (29.1% of annual household expenditure) across all countries, including $397 (16.7%) in Botswana; $590 (37.5%) in Malawi; $230 (7.6%) in South Africa; $578 (38.6%) in Uganda; and $592 (64.2%) in Zimbabwe (Figure 1A). Only 4% (21/581) had access to private healthcare insurance, 12.9% (75/560) had borrowed money, and 11.2% (65/581) had sold possessions to pay for healthcare.

### 3.4 Catastrophic healthcare expenditure

Using a 20% threshold, when combining out‐of‐pocket expenditure and loss of income, 17.9% (145/810, 95% CI 15.3–20.5) of households had already experienced CHE prior to enrolment (Figure 1B and Table S3). This varied from 0.9% (1/106, 95% CI 0.0–2.8%) in South Africa to 27.3% (90/330, 95% CI 22.4–32.1) in Uganda. Among the households of individuals who survived, more than half (50.9%, 296/581, 95% CI 46.9–55.0) had experienced CHE by the end of the trial and this ranged from 16.0% (13/81, 95% CI 7.9–24.2) in South Africa to 68.1% (156/229, 95% CI 62.0–74.2) in Uganda.

### 3.5 Sensitivity analyses

Using a threshold of 10%, 32.7% (265/810, 95% CI 29.5–35.9) of households experienced CHE prior to enrolment (Figure 1C and Table S3) and 67.9% (395/581, 95% CI 64.2–71.7) of survivor households experienced CHE. The proportion experiencing CHE was highest in Uganda at 47.3% (156/330, 95% CI 41.9–52.6) and 86.0% (197/229, 95% CI 81.5–90.5) at each time point.

With regard to gender (Table S4), despite no significant difference between genders at the point of enrolment, we found that among those who survived, out‐of‐pocket expenditure plus lost income was significantly higher among men ($633 vs. $334, *p* = 0.0310), as were the proportions experiencing CHE at the 10% (71% vs. 63%, *p* = 0.0390) and 20% (55% vs. 45%, *p* = 0.0202) thresholds. With regard to the treatment arm (Table S5), prior to enrolment, a larger proportion of those who were randomized to the AMBITION‐cm intervention experienced CHE at the 10% (38% vs. 28%, *p* = 0.0029) and 20% thresholds (22% vs. 14%, *p* = 0.0032) compared to those in the control arm. This difference was not maintained among those who survived at the 10% (69% vs. 67%, *p* = 0.6699) and 20% (54% vs. 48%, *p* = 0.1751) CHE thresholds.

A roughly equal proportion of participants in Zimbabwe had paid for their care in USD (49% [35/72]) and ZWD (51% [37/72]). At the point of enrolment, when combining out‐of‐pocket expenses and lost income, the economic impact on those using US dollars was $64 versus $117 for those using Zimbabwean dollars and CHE was 9% (3/35) and 24% (9/28), respectively. At the end of the trial, the economic impact was $243 and $888, and CHE was experienced by 50% (11/22) and 62% (16/26) of households, respectively.

## 4 DISCUSSION

In this multi‐country study examining the household economic impact of HIV‐associated cryptococcal meningitis, we found more than half of the households of individuals who survived for 10 weeks experienced CHE at a 20% threshold. The incidence of CHE varied across country settings and was highest in Malawi and Uganda. Even at the point of hospitalization, 18% of households had already experienced CHE. These data highlight the profound financial impact of this infection.

Our previous within‐trial analysis found the average healthcare provider cost of treating someone with cryptococcal meningitis with the AMBITION‐cm regimen was $1379 compared to $1237 with the control arm, and that regimen was highly cost‐effective with incremental cost‐effectiveness ratios of $71 in Botswana ranging to $121 in Uganda per life‐year saved [16]. In this analysis, we found the average household economic impact of cryptococcal meningitis was $516 and ranged from $230 in South Africa to $592 in Zimbabwe. In Malawi and Uganda which are low‐income countries, this economic impact was 92% and 60% of Gross Domestic Product per capita, respectively.

Overall, we found that 51% of the households of participants who survived to 10 weeks experienced CHE. This is comparable to findings from WHO national surveys of TB patients from 29 countries which identified a pooled estimate of 49% of all TB patients experiencing CHE, using the same 20% threshold [22], although the only country represented in both these studies is Uganda. This is despite the shorter time‐course in our study compared to TB therapy—10 weeks versus a typical treatment duration of at least 6 months—but cryptococcal meningitis being a more acute infection always requiring hospitalization [23]. The AMBITION‐cm trial covered costs associated with hospitalization and post‐discharge care up to 10 weeks. In settings where inpatient and outpatient HIV care are provided free‐of‐charge, there are frequently additional out‐of‐pocket expenses in the form of user fees to contribute towards, for example, registration, consultation or medication costs [6]. The clinical trial would have enabled participants to avoid some of these costs, which may have cumulatively been significant. It is, therefore, highly likely our findings are underestimations and the economic impact outside of a research setting, including the proportion of households who experience CHE, is far larger. In addition, patients are likely to incur further costs beyond 10 weeks linked to loss of productivity due to the ongoing effects of cryptococcal meningitis and when accessing health services for further follow‐up. As well as being a highly vulnerable time clinically, this financial vulnerability should also be considered, and the role of social protection and support for individuals and households deliberated.

We found that CHE was experienced more by the households of male survivors. This is likely explained by more male survivors being in employment than female survivors and that lost income was only collected at the individual rather than household level. With regard to the treatment arm, despite more of the households of those randomized to the AMBITION intervention experiencing CHE prior to enrolment and before receiving the intervention, this difference was not observed among survivors, suggesting the intervention may have counteracted this random baseline imbalance.

These quantitative data complement previous qualitative methods research by describing numerous healthcare interactions prior to diagnosis [24] and the variation in results across the five country settings are consistent with the experience of the research team. Most participants were the main household earners, reflecting the working age of participants, and most were men [25]. Mortality remains around 25%, even in trials of the best available therapeutics, and individuals with jobs were out of work for a median of more than 10 weeks [12]. HIV‐related illness results in working‐age parents being out of work and has previously been cited as a driving factor for adolescents transitioning out of education and into the workforce to support the home [26]. The economic impact, therefore, extends far beyond the household to the wider society.

The variation in household expenditure observed across country settings was consistent with their overall economic circumstances. For example, food and rent costs were higher in South Africa and Botswana which are upper‐middle‐income countries. The duration of illness was similar across countries apart from in Botswana where individuals were recruited earlier, potentially due to an effective cryptococcal antigen screening programme. The number of previous healthcare interactions prior to hospitalization was a median of one, but this may be an underestimate. In our qualitative methods work in Botswana and Uganda with a purposively selected sample of participants, we found participants had often visited multiple healthcare facilities in the days prior to hospitalization [11]. It may be that recall bias due to the severe nature of the illness led to under‐reporting, further emphasizing that the costs reported in this study are likely to be underestimates.

Out‐of‐pocket expenditure in the 4 weeks up to enrolment was highest in Uganda and Malawi which was due to higher costs of accessing outpatient healthcare, including in the public sector, for example by having to pay for consultations or medication. This partially explains why the overall economic impact and CHE were lower in Botswana and South Africa where public healthcare is more comprehensive and services are provided free at the point of delivery. We also found in Malawi, Uganda and Zimbabwe there was much higher utilization of partners, family and friends to provide care and support. This is consistent with our observations, including in Uganda and Malawi where caregivers are actively encouraged to remain by the bedside to assist with feeding, medication administration and personal care [27]. We did not attribute a cost to this time which would have further accentuated our findings.

The sensitivity analysis exploring the impact of the form of currency in Zimbabwe, although limited by a small sample size, indicates that the households of individuals who paid in ZWD incurred higher relative costs and experienced higher rates of CHE. Our interpretation may be limited by the use of a single annual inflation rate in the context of significant volatility but could be explained by the users’ socio‐economic status impacting their ability to access USD and the relative lower purchasing power of the ZWD, regardless of the exchange rate.

This was the first study of its kind and was embedded within the largest clinical trial for cryptococcal meningitis ever conducted. However, several limitations should be considered when interpreting the findings. This analysis was conducted within a single trial so the reproducibility of the results may be limited; we aimed to partially overcome this by adopting a multi‐country approach, including countries with a range of income levels and analysing overall and by country. We co‐developed the first health economics questionnaire specific to cryptococcal meningitis with individuals with relevant contextual experience and expertise but this was not externally validated, which would be a valuable next step for future studies. We did not ask participants exactly how much money they earned to calculate their annual income and this decision was made after consultation with individual site research teams. We could not, therefore, calculate the economic impact relative to their annual income, nor make comparisons across income groups, but used their annual household expenditure to calculate CHE, which is consistent with the definition. We prioritized CHE for our primary outcome and further research could explore impoverishment resulting from cryptococcal meningitis. Similarly, we prioritized lost income as a proxy for lost time and productivity. Likewise, we did not collect lost income, annual income or educational level of caregivers, and, therefore, could not calculate the secondary opportunity cost associated with care given to participants. Cryptococcal meningitis is a severe neurological infection, and it is likely there will have been some recall bias, particularly in cases where participants were confused for a prolonged period, and we collected data from relatives who may not have been fully aware of the costs incurred.

## 5 CONCLUSIONS

In conclusion, we found the household economic impact of cryptococcal meningitis was an average of $516 per person and that more than half of survivors experienced CHE. It is likely that these figures are higher outside of the research setting. This work highlights the profound financial impact of this devastating infection, the urgent need to prevent individuals from developing cryptococcal meningitis, and provides a rationale to offer financial support and social protection to those affected.

## COMPETING INTERESTS

TSH was a recipient of an investigator award to his institution from Gilead Sciences, speaker fees from Pfizer and Gilead Sciences, and serves as an advisor for F2G. JNJ and GM both declare speaker fees from Gilead Sciences. There are no additional interests declared.

## AUTHORS’ CONTRIBUTIONS

All authors conceptualized the work, developed the methodology and contributed to project administration, data collection, and curation. DSL, TBC, SFM, NY, SJ, TSH and JNJ developed the software. DSL, CM, TBC and NY verified the data. DSL and NY had access to the raw data. DSL, CM, JA and BN analysed the data, validated the results, and created the visualizations and TSH, LC, and JNJ supervised. DSL, CM, JA, BN, TSH, LC and JNJ wrote the original manuscript, and all authors reviewed and edited the manuscript. DSL, CM, LC and JNJ had final responsibility for the decision to publication. SJ, TSH, JNH and LWN acquired the funding.

## FUNDING

Funded by a grant through the European Developing Countries Clinical Trials Partnership (EDCTP) supported by the Swedish International Development Cooperation Agency (SIDA) (TRIA2015‐1092), and the U.K. Department of Health and Social Care, the U.K. Foreign Commonwealth and Development Office, the U.K. Medical Research Council, and Wellcome Trust, through the Joint Global Health Trials scheme (MR/P006922/1). This work was also funded by the National Institute for Health Research (NIHR) through a Global Health Research Professorship to JNJ (RP‐2017‐08‐ST2‐012) using UK aid from the UK Government to support global health research. CM was supported by a Wellcome Trust International Masters Fellowship (212638/Z/18/Z). GM was supported by the Wellcome Trust (098316, 214321/Z/18/Z and 203135/Z/16/Z), and the South African Research Chairs Initiative of the Department of Science and Technology and the National Research Foundation (NRF) of South Africa (Grant No. 64787). The AmBisome was donated by Gilead Sciences Inc. For the purpose of open access, the author has applied a CC BY public copyright license to any Author Accepted Manuscript version arising from this submission. The trial funders, suppliers and drug manufacturers had no role in the design of the trial and this economic analysis; in the collection, analysis or interpretation of the data; or in the preparation of the manuscript or the decision to submit it for publication.

## DISCLAIMER

The views expressed in this publication are those of the author(s) and not necessarily those of the funders.

## ETHICS APPROVAL

The protocol was approved by research ethics committees at the London School of Hygiene and Tropical Medicine, Botswana Ministry of Health and Wellness, Malawi National Health Sciences, University of Cape Town, Uganda National Council for Science and Technology, and Zimbabwe Medical Research Council.

## PATIENT CONSENT

Written informed consent was obtained from participants or from the next‐of‐kin if participants were incapable of consenting because of the clinical condition. If a participant recovered the capacity to provide consent, written informed consent was obtained from that participant and they were free to leave the study if they wished without impacting on their treatment.

## Supporting information




**Table S1**: Summary of economic data collected from AMBITION‐cm trial participants
**Table S2**: Direct and indirect costs incurred due to cryptococcal meningitis during the ten‐week trial period
**Table S3**: Catastrophic healthcare expenditure
**Table S4**: Catastrophic healthcare expenditure, by gender
**Table S5**: Catastrophic healthcare expenditure, by treatment arm

## Data Availability

Anonymized, individualized participant data, a data dictionary and data collection tools are available upon request from the London School of Hygiene and Tropical Medicine Data Compass at https://datacompass.lshtm.ac.uk
