# Supplementary material for: Household economic impact of HIV‐associated cryptococcal meningitis in five countries in Southern and Eastern Africa
Source: J Int AIDS Soc. 2025 Jun 5;28(6):e26441. doi: 10.1002/jia2.26441 (PMC12141757; doi:10.1002/jia2.26441)
Supplement: Supplementary file 1 — Table S1: Summary of economic data collected from AMBITION‐cm trial participants Table S2: Direct and indirect costs incurred due to cryptococcal meningitis during the ten‐week trial period Table S3: Catastrophic healthcare expenditure Table S4: Catastrophic healthcare expenditure, by gender Table S5: Catastrophic healthcare expenditure, by treatment arm [file JIA2-28-e26441-s001.pdf]

Household economic impact of HIV-associated cryptococcal meningitis in five countries in Southern and Eastern Africa

SUPPLEMENTARY MATERIAL

The AMBITION Study Group

In addition to the named authors, the following were members of the Ambition Study Group:

Botswana Harvard AIDS Institute Partnership / Princess Marina Hospital, Gaborone, Botswana – J Goodall, K Lechiile, N Mawoko, T Mbangiwa, J Milburn, R Mmipi, P Ponatshego, I Rulaganyang, K Seatla, K Siamisang, N Tlhako and K Tsholo.

University of Cape Town / Mitchells Plain Hospital / Khayelitsha District Hospital, Cape Town, South Africa – S April, F Bango, A Bekiswa, L Boloko, H Bookholane, T Crede, L Davids, R Goliath, S Hlungulu, R Hoffman, H Kyepa, N Masina, D Maughan, T Mnguni, S Moosa, T Morar, M Mpalali, J Naude, I Oliphant, A Singh, S Sayed, L Sebesho, M Shey, S Shamu and L Swanepoel.

Malawi-Liverpool-Wellcome Trust Clinical Research Programme / Queen Elizabeth Central Hospital, Blantyre, Malawi – M Chasweka, W Chimang’anga, T Chimphambano, E Gondwe, H Mzinganjira, A Kadzilimbile, S Kateta, E Kossam, C Kukacha, B Lipenga, J Ndaferankhande, M Ndalama, R Shah, A Singini, K Stott and A Zambasa.

UNC Project, Kamuzu Central Hospital, Lilongwe, Malawi – T Banda, T Chikaonda, G Chitulo, L Chiwoko, N Chome, M Gwin, T Kachitosi, B Kamanga, M Kazembe, E Kumwenda, M Kumwenda, C Maya, W Mhango, C Mphande, L Msumba, T Munthali, D Ngoma, S Nicholas, L Simwinga, A Stambuli, G Tegha and J Zambezi.

Infectious Diseases Institute / Kiruddu General Hospital, Kampala, Uganda – C Ahimbisibwe, A Akampurira, A Alice, F Cresswell, J Gakuru, E Kagimu, J Kasibante, D Kiiza, J Kisembo, R Kwizera, F Kugonza, E Laker, T Luggya, A Lule, A Musubire, R Muyise, O Namujju, J Ndyetukira, L Nsangi, M Okirwoth, J Rhein, M K Rutakingirwa, A Sadiq, K Ssebambulidde, K Tadeo, A Tukundane and D Williams.

Infectious Diseases Institute / Mbarara Regional Referral Hospital, Mbarara, Uganda – L Atwine, P Buzaare, M Collins, N Emily, C Inyakuwa, S Kariisa, J Mwesigye, S Niwamanya, A Rodgers, J Rukundo, I Rwomushana, M Ssemusu and G Stead.

University of Zimbabwe / Parirenyatwa General Hospital, Harare, Zimbabwe – K Boyd, S Gondo, P Kufa, E Makaha, C Moyo, T Mtisi, S Mudzingwa, C Mutata, T Mwarumba and T Zinyandu.

Institut Pasteur, Paris, France – A Alanio, F Dromer, O Lortholary and A Sturny-Leclere.

London School of Hygiene and Tropical Medicine, London, UK – P Griffin and S Hafeez.

St George’s University, London, UK – A Loyse.

Liverpool School of Tropical Medicine, Liverpool UK – T Chen, T Shiri, E van Widenfelt.

Table S1: Summary of economic data collected from AMBITION-cm trial participants

**BASELINE**	**Participant Demographics**
	Gender<br>Age<br>Years in education<br>Highest qualification<br>Occupation<br>If participant was main earner in the household<br>If not, education level and occupation of main earner
	**Household Expenditure**
	Weekly food expenditure<br>Monthly rent and utilities expenditure<br>Annual large item expenditures (e.g. furniture, electronics, vehicles)
	**Direct costs of illness prior to enrolment**
	Personal expenditure in four weeks up to hospitalisation<br>Expenditure of others in four weeks prior to hospitalisation<br>Previous healthcare interactions (maximum of three)<br>• Location<br>• Provider<br>• Cost of consultation, medication, travel<br>• Time taken
	**Indirect costs of illness prior to enrolment**
	Duration of illness<br>Primary activity missed due to illness<br>Lost income<br>Time others had taken off work to provide care
	**Payment strategies prior to enrolment**
	Access to and use of private medical insurance<br>Borrowing money<br>Selling possessions
**WEEK 10**	**Costs from enrolment to end of trial**
	Duration of illness<br>Primary activity missed due to illness<br>Lost income<br>Out-of-pocket expenditure during trial<br>Payment strategies

Table S2: Direct and indirect costs incurred due to cryptococcal meningitis during the ten-week trial period

**In the ten weeks during the trial:**	**Overall**	**Botswana**	**Malawi**	**South Africa**	**Uganda**	**Zimbabwe**
	**n = 581**	**n = 60**	**n = 163**	**n = 81**	**n = 229**	**n = 48**
Duration of illness in days during trial 10 weeks<br>Median (IQR)<br>Mean (SD)	70 (42-70)<br>56 (19.9)	62 (42-70)<br>54.1 (19.5)	70 (30-70)<br>52.8 (23.4)	70 (70-70)<br>66.6 (12.3)	70 (49-70)<br>57.5 (17.2)	47 (25-70)<br>44.3 (21.3)
Primary activity missed due to illness (n(%))						
Working	455 (78.3%)	49 (81.7%)	125 (76.7%)	34 (42.0%)	206 (90.0%)	41 (85.4%)
Studying	16 (2.8%)	0 (0%)	9 (5.5%)	1 (1.2%)	5 (2.2%)	1 (2.1%)
Maintaining the House	15 (2.6%)	1 (1.7%)	4 (2.5%)	3 (3.7%)	5 (2.2%)	2 (4.2%)
Caring for Children	23 (4.0%)	1 (1.7%)	12 (7.4%)	3 (3.7%)	6 (2.6%)	1 (2.1%)
Nothing	72 (12.4%)	9 (15.0%)	13 (8.0%)	40 (49.4%)	7 (3.1%)	3 (6.3%)
If working, days spent off work during trial	**n=455**	**n=49**	**n=125**	**n=34**	**n=206**	**n=41**
Median (IQR)	63 (38-70)	60 (42-70)	70 (28-70)	70 (50-70)	70 (40-70)	45 (21-67)
Mean (SD)	52.8 (20.8)	53 (18.4)	50.1 (23.9)	56.1 (21.1)	55.9 (18.2)	42.2 (22.5)
If working, lost income during trial (n(%))	**n=455**	**n=49**	**n=125**	**n=34**	**n=206**	**n=41**
Yes	326 (71.7%)	25 (51%)	58 (46.4%)	22 (64.7%)	191 (92.7%)	30 (73.2%)
No	129 (28.3%)	24 (49%)	67 (53.6%)	12 (35.3%)	15 (7.3%)	11 (26.8%)
If lost income, income lost during trial (Mean (SD)) USD	**n=326**<br>558.97 (2064.25)	**n=25**<br>720.01 (715.72)	**n=58**<br>967.25 (4639.61)	**n=22**<br>580.65 (516.78)	**n=191**<br>383.36 (505.25)	**n=30**<br>737.56 (1600.71)
Days others spent providing care during trial Median (IQR) USD<br>Mean (SD) USD	**n=581**<br>14 (0-20)<br>17.8 (21.2)	**n=60**<br>0 (0-2)<br>3.0 (10.5)	**n=163**<br>20 (10-42)<br>27.7 (24.4)	**n=81**<br>0 (0-0)<br>0.3 (1.7)	**n=229**<br>14 (14-21)<br>22.4 (20.5)	**n=48**<br>10 (7-14)<br>11.1 (5.7)
Additional out of pocket expenditure during trial (Mean (SD)) USD	**n=581**<br>72.81 (126.30)	**n=60**<br>28.59<br>(82.89)	**n=163**<br>125.17 (136.53)	**n=81**<br>6.97<br>(25.68)	**n=229**<br>73.79 (139.59)	**n=48**<br>56.70 (139.59)
Overall out of pocket expenditure plus lost income (Mean (SD)) USD	**n=581**<br>386.45 (1592.38)	**n=60**<br>328.59 (593.38)	**n=163**<br>469.34 (2818.93)	**n=81**<br>164.67 (373.66)	**n=229**<br>393.54 (528.35)	**n=48**<br>517.68<br>(1318.01)

Table S3: Catastrophic healthcare expenditure

	Overall	Botswana	Malawi	South Africa	Uganda	Zimbabwe
**All prior to trial enrolment**	**n=810**	**n=73**	**n=230**	**n=106**	**n=330**	**n=71**
Estimated annual household expenditure USD	1716.93 (1939.14)	2713.10 (2072.41)	1561.05 (1918.62)	2863.13 (2744.96)	1414.08 (1516.95)	894.04 (810.42)
Out of pocket expenditure plus lost income USD	132.44 (250.02)	114.87 (280.04)	123.53 (231.25)	59.81 (134.97)	174.84 (291.38)	90.74 (147.07)
Out of pocket expenditure as proportion of annual household expenditure (%)	7.71	4.23	7.91	2.09	12.36	10.15
Catastrophic Healthcare Expenditure (10%) n(%)	265 (32.72)	9 (12.33)	71 (30.87)	5 (4.72)	156 (47.27)	24 (33.80)
Catastrophic Healthcare Expenditure (20%) n(%)	145 (17.90)	3 (4.11)	39 (16.96)	1 (0.94)	90 (27.27)	12 (16.90)
**Survived to 10 weeks**	**n = 581**	**n = 60**	**n = 163**	**n = 81**	**n = 229**	**n = 48**
Estimated annual household expenditure USD	1775.31 (1948.48)	2369.31 (1576.40)	1573.98 (1721.80)	3019.25 (2958.75)	1501.73 (1666.96)	922.54 (876.06)
Out of pocket expenditure plus lost income USD	516.08 (1633.16)	396.58 (646.44)	589.97 (2834.74)	230.15 (488.37)	579.99 (682.53)	592.12 (1349.98)
Out of pocket expenditure as proportion of annual household expenditure (%)	29.07	16.74	37.48	7.62	38.62	64.19
Catastrophic Healthcare Expenditure (10%) n(%)	395 (67.99)	23 (38.33)	116 (71.17)	21 (25.93)	197 (86.03)	38 (79.17)
Catastrophic Healthcare Expenditure (20%) n(%)	296 (50.95)	18 (30.00)	82 (50.31)	13 (16.05)	156 (68.12)	27 (56.25)
**GDP per capita (2022)**		7726.11	643.43	6766.48	964.35	1676.82
Prior to trial expenditure and lost income as proportion of GDP (%)		1.49	19.20	0.88	18.13	5.41
Survived to 10 weeks expenditure and lost income as proportion of GDP (%)		5.13	91.69	3.40	60.14	35.31

Table S4: Catastrophic healthcare expenditure, by gender

	**Overall**	**Overall Male**	**Overall Female**	**P value**
**All prior to trial enrolment**	**n=810**	**n=491**	**n=319**	
Estimated annual household expenditure USD	1716.93 (1939.14)	1758.79 (1837.34)	1652.51 (2087.48)	p=0.4463
Out of pocket expenditure plus lost income USD	132.44 (250.02)	140.03 (255.69)	120.77 (240.95)	p=0.2843
Catastrophic Healthcare Expenditure (10%) n(%)	265 (32.72)	173 (35.23)	92 (28.84)	p=0.0582
Catastrophic Healthcare Expenditure (20%) n(%)	145 (17.90)	93 (18.95)	52 (16.30)	p=0.3389
**Survived to 10 weeks**	**n = 581**	**n=354**	**n=227**	
Estimated annual household expenditure USD	1775.31 (1948.48)	1821.95 (1840.93)	1702.57 (2107.41)	p=0.4717
Out of pocket expenditure plus lost income USD	516.08 (1633.16)	633.06 (2041.13)	333.65 (531.73)	p=0.0310*
Catastrophic Healthcare Expenditure (10%) n(%)	395 (67.99)	252 (71.19)	143 (63.00)	p=0.0390*
Catastrophic Healthcare Expenditure (20%) n(%)	296 (50.95)	205 (54.80)	102 (44.93)	p=0.0202*

Table S5: Catastrophic healthcare expenditure, by treatment arm

	Overall	AMBITION-cm Regimen	Control	P value
**All prior to trial enrolment**	**n=810**	**n=413**	**n=397**	
Estimated annual household expenditure USD	1716.93 (1939.14)	1690.18 (2004.78)	1744.76 (1870.53)	p=0.6891
Out of pocket expenditure plus lost income USD	132.44 (250.02)	145.32 (272.85)	119.05 (223.37)	p=0.1350
Catastrophic Healthcare Expenditure (10%) n(%)	265 (32.72)	155 (37.53)	110 (27.71)	p=0.0029
Catastrophic Healthcare Expenditure (20%) n(%)	145 (17.90)	90 (21.79)	55 (13.85)	p=0.0032
**Survived to 10 weeks**	**n = 581**	**n=298**	**n=283**	
Estimated annual household expenditure USD	1775.31 (1948.48)	1788.61 (2030.82)	1761.30 (1861.33)	p=0.8660
Out of pocket expenditure plus lost income USD	516.08 (1633.16)	458.22 (670.16)	577.01 (2237.29)	p=0.3813
Catastrophic Healthcare Expenditure (10%) n(%)	395 (67.99)	205 (68.79)	190 (67.14)	p=0.6699
Catastrophic Healthcare Expenditure (20%) n(%)	296 (50.95)	160 (53.69)	136 (48.06)	p=0.1751
